# Cell-Free Circulating Mitochondrial DNA: A Potential Blood-Based Marker for Atrial Fibrillation

**DOI:** 10.3390/cells9051159

**Published:** 2020-05-08

**Authors:** Marit Wiersma, Denise M.S. van Marion, Emma J. Bouman, Jin Li, Deli Zhang, Kennedy S. Ramos, Eva A.H. Lanters, Natasja M.S. de Groot, Bianca J.J.M. Brundel

**Affiliations:** 1 Department of Physiology, Amsterdam UMC, Vrije Universiteit Amsterdam, Amsterdam Cardiovascular Sciences, 1081 HV Amsterdam, The Netherlands; d.vanmarion@amsterdamumc.nl (D.M.S.v.M.); e.j.bouman@student.vu.nl (E.J.B.); j.li@amsterdamumc.nl (J.L.); d.zhang@amsterdamumc.nl (D.Z.); k.silvaramos@amsterdamumc.nl (K.S.R.); 2 Netherlands Heart Institute, 3511 EP Utrecht, The Netherlands; 3 Department of Cardiology, Erasmus Medical Center, 3015 DG Rotterdam, The Netherlands; e.lanters@erasmusmc.nl (E.A.H.L.); n.m.s.degroot@erasmusmc.nl (N.M.S.d.G.)

**Keywords:** atrial fibrillation, biomarker, mitochondrial DNA, serum

## Abstract

Atrial fibrillation (AF), the most common, progressive tachyarrhythmia is associated with serious complications, such as stroke and heart failure. Early recognition of AF, essential to prevent disease progression and therapy failure, is hampered by the lack of accurate diagnostic serum biomarkers to identify the AF stage. As we previously showed mitochondrial dysfunction to drive experimental and human AF, we evaluated whether cell-free circulating mitochondrial DNA (cfc-mtDNA) represents a potential serum marker. Therefore, the levels of two mtDNA genes, COX3 and ND1, were measured in 84 control patients (C), 59 patients undergoing cardiac surgery without a history of AF (SR), 100 paroxysmal (PAF), 116 persistent (PeAF), and 20 longstanding-persistent (LS-PeAF) AF patients undergoing either cardiac surgery or AF treatment (electrical cardioversion or pulmonary vein isolation). Cfc-mtDNA levels were significantly increased in PAF patients undergoing AF treatment, especially in males and patients with AF recurrence after AF treatment. In PeAF and LS-PeAF, cfc-mtDNA levels gradually decreased. Importantly, cfc-mtDNA in serum may originate from cardiomyocytes, as in vitro tachypaced cardiomyocytes release mtDNA in the medium. The findings suggest that cfc-mtDNA is associated with AF stage, especially in males, and with patients at risk for AF recurrence after treatment.

## 1. Introduction

Atrial fibrillation (AF) is the most common progressive cardiac tachyarrhythmia globally. AF is present in 3% of the total population, with a higher prevalence in the elderly, and is associated with serious complications such as stroke, heart failure, impaired cognitive function, and increased mortality [1,2,3]. Early recognition of AF is essential to prevent disease progression from recurrent intermitted episodes to finally the permanent AF stage. This progression is accompanied by a gradual increase in therapy failure and the end-stage can, even with extensive therapy, not be treated [1,2,3]. Compared to age-matched males, women have a higher risk of AF-related complications, including mortality and stroke. Women also have higher recurrence rates after cardioversion or ablative therapy and experience more side effects of pharmacological therapy [4].

At present, AF can only be diagnosed with a surface electrocardiogram when a patient already suffers from AF. In addition, this rhythm registration cannot assess the stage of AF, which is essential for selection of the appropriate therapy. Hence, early recognition of AF and the start of effective treatment is seriously hampered. Therefore, there is an urgent need to develop successful diagnostic biomarkers for AF [2].

The use of serum biomarkers is an interesting approach to assess (patho)physiological processes and therapeutic responses. Serum biomarkers are used in the identification and management of several diseases, including heart failure [5], myocardial infarction [6], cancer [7], and diabetes [8]. So far, no specific serum biomarkers to assess AF stage and to predict the outcome of AF treatment are available. Nevertheless, several AF serum biomarkers, such as troponin (myocardial injury) [2], brain natriuretic peptide (cardiovascular stress) [2,9], creatinine (renal dysfunction) [10], C-reactive protein (inflammation) [11], and fibroblast growth factor 23 [9], have been associated with AF pathology. Only in 2016, the European Society of Cardiology implemented the use of troponin and natriuretic peptide into their guidelines, to predict stroke and bleeding risk in AF patients [2].

Cell-free circulating mitochondrial DNA (cfc-mtDNA) is successfully used as a biomarker for conditions associated with mitochondrial dysfunction or stress, such as cancer progression [12,13], dengue severity [14], cardiac arrest survival [15], and diabetes mellitus [16]. Cfc-mtDNA acts as a damage-associated pattern (DAMP) [17], causing inflammation, which might be associated with the inflammation seen in AF patients [18,19,20] and an experimental mouse model for AF [20]. We recently observed mitochondrial dysfunction to underlie experimental and human AF [21]. As mitochondrial dysfunction may initiate the release of cfc-mtDNA in the circulation [22], we sought to evaluate the value of cfc-mtDNA as a blood-based marker for AF stage, sex differences, and AF recurrence after treatment, in a population of patients undergoing AF treatment (electrical cardioversion (ECV) or pulmonary vein isolation (PVI)) or cardiac surgery.

## 2. Materials and Methods

### 2.1. Patient Material

Blood samples were collected from control patients, who either had Wolff–Parkinson–White syndrome, premature ventricular beats, or were persons with a family history of Brugada syndrome (referred for Ajmaline testing), and from AF and sinus rhythm (SR) patients prior to scheduled intervention (open-heart surgery, ECV or PVI) in BD^TM^ Vacutainer^TM^ SST^TM^ II Advance Tubes (Thermo Fisher Scientific, Amsterdam, The Netherlands) as described in the Halt and Reverse study (MEC 2014-393) [23]. Serum was collected after centrifugation at 2000× *g* for 10 min at 4 °C and stored at −80 °C. All patients signed written informed consent prior to inclusion. This sub-study is part of the HALT & REVERSE trial (MEC-2014-393) and was approved by the institutional medical ethical committee. This study is carried out according to the principals of the Declaration of Helsinki and in accordance with the Medical Research Committee involving Human Subjects Act.

### 2.2. HL-1 Cardiomyocyte Culture and Tachypacing

HL-1 mouse atrial cardiomyocytes were obtained from Dr. Claycomb (Louisiana State University, New Orleans, LA, USA) [24] and were maintained in full Claycomb medium (Sigma, Zwijndrecht, The Netherlands) supplemented with FBS (10%, PAA Laboratories GmbH, Pasching, Austria), penicillin (100 U/mL, Gibco, Landsmeer, The Netherlands), streptomycin (100 µg/mL, Gibco), L-glutamine (4 mM, Gibco), L-ascorbic acid (0.3 mM, Sigma), and norepinephrine (100 µM, Sigma). The HL-1 cardiomyocytes were grown on cell culture plastics coated with 0.02% gelatin (Sigma) at 37 °C in 5% CO_2_.

The cardiomyocytes were subjected to tachypacing at 6 Hz, 40 V, and 20 ms for 2, 4, 6, and 8 h utilizing the C-Pace100^TM^-culture pacer (IonOptix Corporation, Amsterdam, The Netherlands). Control cardiomyocytes were subjected to their endogenous rate, 1 Hz, 40 V, and 20 ms. After normal or tachypacing, medium was collected, spun down to remove floating cells, and DNA was isolated as described below.

### 2.3. DNA Isolation and Quantitative PCR

Total DNA was isolated from 200 µL medium from normal- or tachypaced HL-1 cardiomyocytes or 50 µL control patient/AF patient serum in 150 µL phosphate buffered saline (PBS) utilizing the Nucleospin Tissue kit (Macherey-Nagel, Landsmeer, The Netherlands) according to manufacturer’s instructions. Isolated DNA was used to determine DNA levels (a.u.) utilizing the CFX384 Real-time system C1000 Thermocycler (Bio-Rad, Lunteren, The Netherlands) in combination with SYBR green Supermix (Bio-Rad). Briefly, DNA, SYBR green Supermix and 10 µM forward and reverse primer-mix (Invitrogen, The Netherlands, Table 1) were added in a 384-well PCR plate (Bio-Rad) in triplicate per sample. Thermal cycling conditions were performed as a two-step approach using a pre-denaturating step at 50 °C for 2 min and 95 °C for 10 min, followed by 40 cycles of 95 °C for 15 s and 60 °C for 1 min with data collection, ending with a melting curve analysis continuously from 60 °C to 95 °C. Mitochondrial DNA levels were adjusted for nuclear DNA levels (18S rRNA) [25] and analyzed using the ΔC_T_ method.

### 2.4. HSP60 ELISA

HSP60 concentration in serum of control patients and AF patients was determined utilizing the Human Total HSP60 DuoSet IC ELISA kit (R&D Systems, Abingdon, UK), according to the manufacturer’s instructions.

### 2.5. Mitochondrial Dysfunction Measurements in HL-1 Atrial Cardiomyocytes

#### 2.5.1. ATP Measurements

HL-1 atrial cardiomyocytes were lysed and homogenized in 1/20 part 1.5% trichloroacetic acid. Then, one part Tris-buffer (pH 8.0) supplemented with 1 mM sodium fluoride was added, according to the protocol of Promega (ENLITEN ATP assay system bioluminescence detection for ATP measurement—instructions for the use of product FF2000). ATP levels were measured utilizing the ATP Bioluminescence Assay Kit CLSII (Roche, Almere, The Netherlands), according to the manufacturer’s instructions. Briefly, samples and luciferase reagent (supplied) were added at a 1:1 ratio into a white 96-well plate, and ATP levels were measured by bioluminescence utilizing the Mithras LB 940 Multimode Microplate Reader (Berthold Technologies, Bad Wildbad, Germany).

#### 2.5.2. Mitochondrial Membrane Potential Analysis

HL-1 atrial cardiomyocytes were incubated with 100 nM TMRE (ab113852, Abcam, Cambridge, UK) and 100 nM Mitotracker Deep Red (Life Technologies, BleisWijk, The Netherlands) in DMEM for 20 min at 37 °C. The cardiomyocytes were then washed 1x with DMEM, 1x with phosphate buffered saline (PBS); then complete Claycomb medium was added. Live images were obtained by the Zeiss Axiovert 200 M Marianas^TM^ digital imaging inverted microscope system, utilizing a 16-bit cooled charge-coupled device camera (Cooke SensiCam SVGA, Auburn Hills, MI, USA) with Cy3 and CY5 filter blocks and a 63×-oil objective and Slidebook^TM^ (Intelligent Imaging Innovations Inc., Denver, CO, USA) to control hardware and view images. Mitochondrial membrane potential was analyzed of ten random fields, containing at least 10 cardiomyocytes, utilizing ImageJ software (v1.49, NIH, Washington D.C., USA). For analysis, the grey intensity and cell area were measured for each separate cardiomyocyte for both TMRE and Mitotracker Deep Red, of which background grey intensity was subtracted. TMRE values were divided by Mitotracker Deep Red values and adjusted for cell area.

#### 2.5.3. Mitochondrial Morphology Analysis

HL-1 atrial cardiomyocytes were incubated with 100 nM Mitotracker Deep Red (Life Technologies) in DMEM (Gibco) for 30 min at 37 °C. The cardiomyocytes were thereafter washed twice with DMEM, twice with PBS, and fixated with 4% formaldehyde (Klinipath, Duiven, The Netherlands) for 15 min at 37 °C. The cardiomyocytes were washed again twice with PBS and mounted with Vectashield (Vector Laboratories, Burlingame, CA, USA). Images were obtained by the Zeiss Axiovert 200 M Marianas^TM^ digital imaging inverted microscope system, utilizing a 16-bit cooled charge-coupled device camera (Cooke SensiCam SVGA) with a CY5 filter block and a 63x-oil objective and Slidebook^TM^ (Intelligent Imaging Innovations Inc.) to control hardware and view images. Mitochondrial morphology per single cardiomyocyte of ten random fields containing at least 10 cardiomyocytes was scored by an investigator blinded for the conditions. The mitochondrial network was scored as tubular, intermediate, or fragmented: tubular when it appeared as long, intertwining tubules; intermediate when the tubules were at least 30% shorter and also dots (single mitochondria) were present; and fragmented when >70% of the network consisted of dots instead of tubules. The amount of tubular, intermediate, or fragmented mitochondrial morphology was expressed as percentage of total cardiomyocytes to show the distribution of the morphology between conditions.

#### 2.5.4. Mitochondrial Calcium Transient Measurements

HL-1 cardiomyocytes were incubated for 30 min with 5 µM of Rhod-2 AM (Abcam, Cambridge, UK) at 37 °C in DMEM (Gibco), followed by three times washing with DMEM. Rhod-2 AM-loaded cardiomyocytes were excited by a 600 nm laser with emission at 605 nm and amplitudes were recorded with the Myocyte Calcium and Contractility System (IonOptix Corporation). The live recording of the mitochondrial calcium transients (CaT_mito_), which provides an indication of changes in mitochondrial Ca^2+^, was performed at 1 Hz stimulation (normal pacing) at 37 °C. The relative values of fluorescent signals were determined utilizing the following calculation: Fcal = F1/F0, where F1 is the fluorescent signal at any given time and F0 is the fluorescent signal at rest. Mean values from each experimental condition were based on 7 consecutive CaT_mito_ in at least 25 cardiomyocytes.

#### 2.5.5. Mitochondrial Stress Analysis

Total RNA was isolated from HL-1 cardiomyocytes utilizing the Nucleospin RNA isolation kit (Macherey-Nagel, Landsmeer, The Netherlands). First strand cDNA was generated using the iScript cDNA sysnthesis kit (Bio-Rad) and subsequently used as a template for quantitative real-time PCR. Relative changes in transcription level of the mitochondrial stress markers HSP60 and HSP10 were determined using the CFX384 Real-time system C1000 Thermocycler (Bio-Rad) in combination with SYBR Green Supermix (Bio-Rad). mRNA levels were expressed in relative units on the basis of a standard curve and adjusted for GAPDH levels. Primer pairs utilized are the following: HSP60 fw: TGACTTTGCAACAGTCACCC and rv: GCTGTAGCTGTTACAATGGGG, HSP10 fw: CTCCAACTTTCACACT-GACAGG and rv: GCCGAAACTGTAACCAAAGG and GAPDH fw: CATCAAGAAGGTGGTGAAGC and rv: ACCACCCTGTTGCTGTAG.

### 2.6. Statistical Analysis

Results are expressed as mean ± SEM. Differences in patients’ characteristics were evaluated utilizing a Mann–Whitney test with a Bonferroni correction for categorical variables and a Student’s t-test with a Benjamini–Hochberg correction for continuous variables. Group-mean differences between control patients and patients with different stages of AF were evaluated with Student’s t-test, using the correction for unequal variances when necessary [30], and the Benjamini–Hochberg procedure was used to adjust for multiple testing. Linear correlation was determined by Pearson correlation analysis. A value of *p* ≤ 0.05 was considered statistically significant. SPSS version 22 was used for all statistical evaluations. 

## 3. Results

### 3.1. Characteristics of the Study Population

The control and AF patient’s demographic and clinical characteristics are summarized in Table 2 and Tables S1–S4. An overview scheme and description of classifications are presented in Figure S1. This study included 84 control patients (C), 59 sinus rhythm (SR) patients, 100 paroxysmal AF (PAF), 116 persistent AF (PeAF), and 20 longstanding-persistent AF (LS-PeAF) patients. Control persons had either Wolff–Parkinson–White syndrome, premature ventricular beats, or were persons with a family history of Brugada syndrome (referred for Ajmaline testing). SR patients suffered from underlying cardiovascular disease, such as coronary artery disease, mitral valve disease, and/or aortic valve disease, and underwent open-heart surgery. AF patients were either undergoing open-heart surgery for an underlying cardiovascular disease or were treated specifically for AF by ECV or PVI. Approximately 32% of the AF-treated patients had an ECV/PVI before. In the current study, we distinguished between AF patients undergoing open-heart surgery (cardiac surgery group) or AF treatment (ECV/PVI group). There were significant differences between the control group, the SR group, and the AF stages regarding sex and age, with the SR group being more similar to the AF stages (Table 2). Compared to control persons, SR patients and AF patients in all groups had a higher BMI and had more often hypertension and diabetes mellitus, all common risk factors for AF.

### 3.2. Association Between Cell-Free Circulating mtDNA and AF Stage

**Figure 1:**
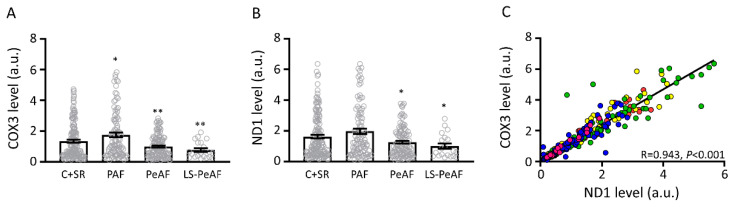
cfc-mtDNA levels are associated with AF stages. Cfc-mtDNA levels for (**A**) cytochrome oxidase 3 (COX3) and (**B**) NADH dehydrogenase subunit 1 (ND1) in C+SR patients and in patients with different AF stages. (**C**) Correlation between COX3 and ND1 levels for all included patients (**C**) red, SR: yellow, PAF: green, PeAF, blue, LS-PeAF: pink). (**C**) control, SR: sinus rhythm PAF: paroxysmal AF, PeAF: persistent AF, LS-PeAF: longstanding-persistent AF. * *p* < 0.05, ** *p* < 0.01 vs. C+SR.

To measure the level of cfc-mtDNA in the serum of all the control patients, SR patients and AF patients (as included in Table 2), two mtDNA genes, cytochrome c oxidase subunit 3 (COX3), and NADH dehydrogenase subunit 1 (ND1) were selected. Due to differences in treatment procedures, we compared cfc-mtDNA levels of AF patients in the ECV/PVI group with control patients (C) and AF patients in the cardiac surgery group with SR patients, as these latest groups underwent cardiac surgery. First, all AF patients were grouped (cardiac surgery and ECV/PVI groups), and cfc-mtDNA levels were compared to grouped control patients and SR patients (C+SR, Figure S2, Table S5). COX3 cfc-mtDNA levels were increased in PAF, and COX3 and ND1 levels were gradually and significantly decreased in PeAF and LS-PeAF compared to C+SR (Figure 1A,B). As expected, COX3 and ND1 levels correlated significantly (Figure 1C).

To determine whether COX3 and ND1 can reliably represent general cfc-mtDNA levels in serum, the levels of eight additional mtDNA genes were determined in 10 individuals per group. These eight additional mtDNA genes included: ATP synthase, subunit 8 (ATP8); cytochrome B (CYB); NADH dehydrogenase, subunit 2 (ND2); NADH dehydrogenase, subunit 3 (ND3); NADH dehydrogenase, subunit 4L (ND4L); NADH dehydrogenase, subunit 4 (ND4); NADH dehydrogenase, subunit 5 (ND5); and NADH dehydrogenase, subunit 6 (ND6). All eight additional mtDNA genes show a trend in increased levels in PAF and a gradual reduction in PeAF and LS-PeAF compared to C (Figure S3). These results suggest that there is an association between the general cfc-mtDNA level and AF stage.

### 3.3. Cfc-mtDNA Levels in Patients Undergoing AF Treatment or Cardiac Surgery 

**Figure 2:**
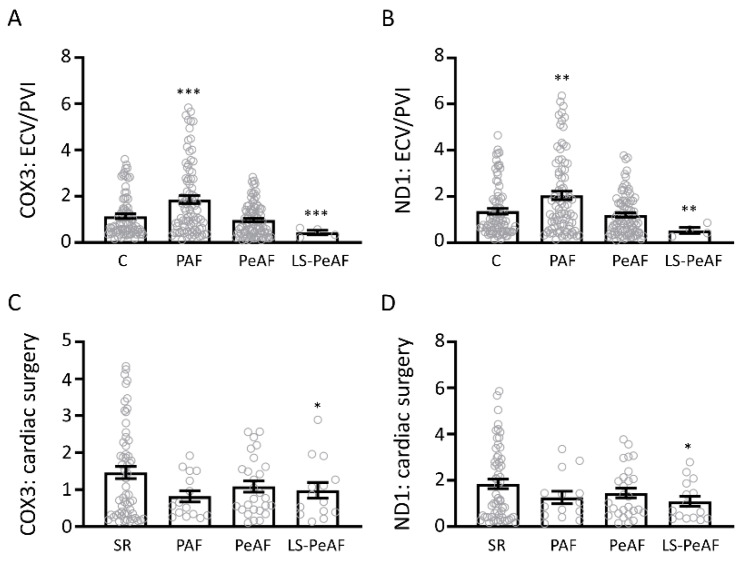
Cfc-mtDNA levels in patients undergoing AF treatment or cardiac surgery. Cfc-mtDNA levels for (**A**) COX3 and (**B**) ND1 in control patients and in patients with different AF stages of the ECV/PVI subgroup. Cfc-mtDNA levels for (**C**) COX3 and (**D**) ND1 in SR patients and in patients with different AF stages of the cardiac surgery subgroup. C: control, SR: sinus rhythm, PAF: paroxysmal AF, PeAF: persistent AF, LS-PeAF: longstanding-persistent AF. * *p* < 0.05, ** *p* < 0.01, *** *p* < 0.001 vs. C or SR.

Next, we determined the cfc-mtDNA levels in patients undergoing AF treatment (ECV/PVI) or cardiac surgery, for which C and SR were used as control population, respectively. Blood samples were taken before AF treatment or cardiac surgery. We found in the AF treatment group a significant increase in cfc-mtDNA levels in PAF and a decrease in LS-PeAF, for both COX3 and ND1, compared to C (Figure 2A,B, Table S5). The cardiac surgery group showed a minor significant difference in cfc-mtDNA levels in LS-PeAF (Figure 2C,D, Table S5). These results suggest that the level of cfc-mtDNA may be a marker in AF patients suffering primarily from AF, while it is less suitable for AF patients suffering from an underlying cardiovascular disease.

### 3.4. Sex Differences in Relation to cfc-mtDNA

**Figure 3:**
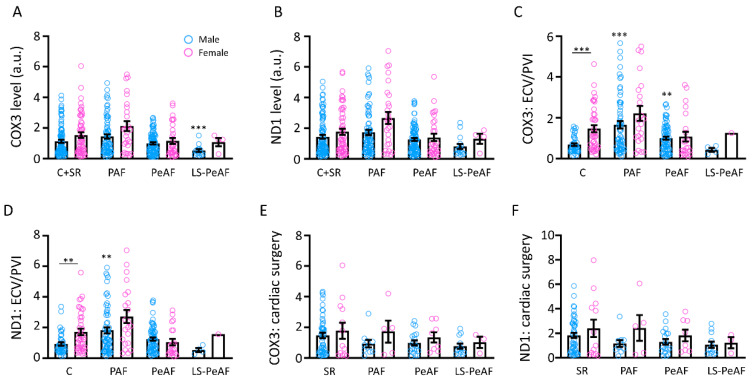
Sex differences in cfc-mtDNA levels. Cfc-mtDNA levels in males and females for (**A**) COX3 and (**B**) ND1 in the total study population; (**C)** COX3 and (**D**) ND1 in control patients and in patients with different AF stages of the ECV/PVI subgroup; and (**E**) COX3 and (**F**) ND1 in SR patients and in patients with different AF stages of the cardiac surgery subgroup. C: control, SR: sinus rhythm, PAF: paroxysmal AF, PeAF: persistent AF, LS-PeAF: longstanding-persistent AF. ** *p* < 0.01, *** *p* < 0.001 vs. C male.

It is widely known that sex differences exist in AF [31]. To study differences in the amount of cfc-mtDNA between male and female patients, COX3 and ND1 levels were compared between the groups. We found in the total study population no differences in cfc-mtDNA levels between males and females, although males show a significant decrease of COX3 in LS-PeAF compared to C+SR (Figure 3A,B). In the ECV/PVI subpopulation, we found that control patients already showed a significant difference in the cfc-mtDNA levels between male and female, with the females having a higher level in serum (Figure 3C,D). Furthermore, cfc-mtDNA was significantly increased in male PAF compared to male C, which was not the case for female PAF compared to female C (Figure 3C,D). No differences were found in the cardiac surgery subgroup (Figure 3E,F). These results implicate that cfc-mtDNA levels are especially associated with male PAF patients suffering primarily from AF.

### 3.5. Cfc-mtDNA Levels May Indicate Recurrence after AF Treatment

**Figure 4:**
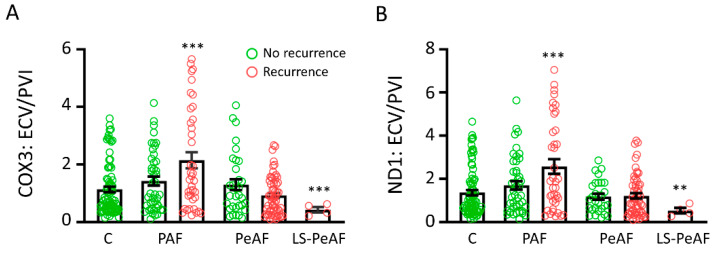
Cfc-mtDNA levels may be used in recurrence prediction in PAF. Cfc-mtDNA levels for (**A**) COX3 and (**B**) ND1 in control patients and in patients with different AF stages of the ECV/PVI subgroup. C: control, PAF: paroxysmal AF, PeAF: persistent AF, LS-PeAF: longstanding-persistent AF. ** *p* < 0.01, *** *p* < 0.001 vs. C.

A high percentage of the ECV/PVI subpopulation showed AF recurrence within 1 year after ECV or PVI treatment, with 58.3% (PAF), 72.1% (PeAF), and 100% (LS-PeAF) for ECV and with 43.8% (PAF), 81.0% (PeAF), and 100% (LS-PeAF) for PVI. To study whether cfc-mtDNA levels are correlated with AF recurrence in both groups, COX3 and ND1 levels were compared in patients with and without AF recurrence. PAF patients with an AF recurrence after treatment showed significant higher cfc-mtDNA levels for both COX3 and ND1 compared to control patients (and borderline significant compared to PAF without an AF recurrence after treatment, *p* = 0.054 for COX3 and *p* = 0.053 for ND1), while LS-PeAF patients with an AF recurrence showed significant lower cfc-mtDNA levels compared to control patients (Figure 4A,B, Figure S4). This effect was especially observed in patients undergoing PVI, as this group contained mainly PAF patients (Figure S4). Interestingly, cfc-mtDNA levels in PAF patients without an AF recurrence after treatment were comparable to control patients. These findings associate the cfc-mtDNA levels, especially of PAF patients, with the risk of recurrence after PVI treatment.

### 3.6. HSP60 Is Not a Mitochondrial Biomarker for AF

**Figure 5:**
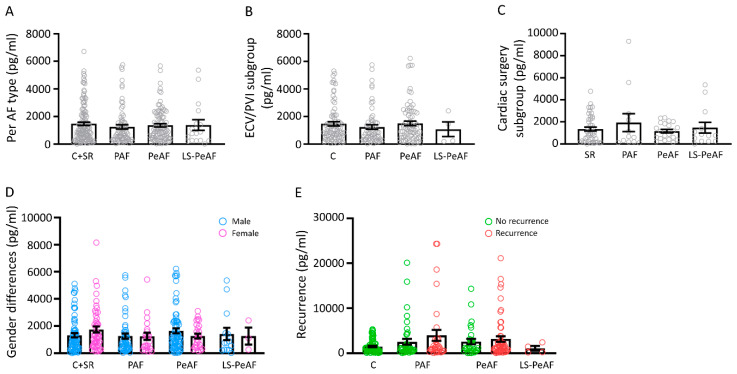
Serum HSP60 is not an applicable biomarker for AF. HSP60 protein levels for (**A**) the total study population, (**B**) patient population of the ECV/PVI subgroup, (**C**) patient population of the cardiac surgery subgroup, (**D**) sex differences and (**E**) AF recurrence. (**C**) control, SR: sinus rhythm, PAF: paroxysmal AF, PeAF: persistent AF, LS-PeAF: longstanding-persistent AF.

Heat shock protein 60 (HSP60), a mitochondrial stress-related chaperone, has been found to act as a biomarker in cancer [32] and heart failure [33]. Therefore, we tested whether HSP60 protein levels could also represent a potential mitochondrial serum marker in AF. However, we did not find any differences in the concentration of serum HSP60 between C+SR patients and patients in the different AF stages (Figure 5A) or between the cardiac surgery and ECV/PVI subgroups (Figure 5B,C). Moreover, there is no difference in expression levels between males and females and in patients without or with AF recurrence (Figure 5D,E). These results show that serum HSP60 is not an applicable blood-based marker for AF.

### 3.7. Mitochondrial DNA Is Released from HL-1 Atrial Cardiomyocytes upon Tachypacing and Is Associated with Mitochondrial Damage

**Figure 6:**
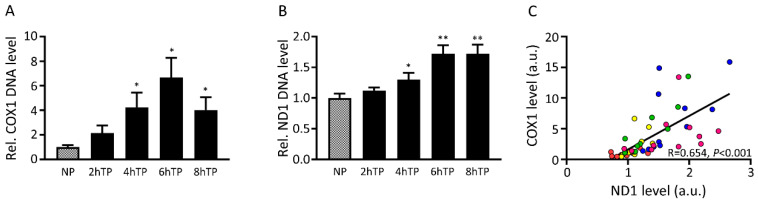
Tachypaced HL-1 cardiomyocytes release mtDNA into the medium. Relative (**A**) cytochrome oxidase 1 (COX1) and (**B**) ND1 expression level in the medium in response to a time-course of tachypacing (TP) compared to normal-paced (NP) HL-1 cardiomyocytes. (**C**) Correlation between COX1 and ND1 levels for all included conditions (NP: red, 2hTP: yellow, 4hTP: green, 6hTP: blue, 8hTP: pink). * *p* < 0.05, ** *p* < 0.01 vs. NP.

As mitochondrial dysfunction may result in the release of cfc-mtDNA in the circulation [22], we examined the release of cfc-mtDNA in the medium of HL-1 atrial cardiomyocytes, subjected to tachypacing to mimic AF [21,34,35,36]. We found a significant increase in cytochrome c oxidase subunit 1 (COX1) and ND1 levels in the medium from 4 h of tachypacing onwards (Figure 6A,B), compared to normal pacing. Moreover, the COX1 and ND1 levels correlated significantly (Figure 6C), just as we observed in serum of the AF patients. This finding suggests that mtDNA is released out of the cardiomyocyte upon rapid electrical stimulation. Therefore, the observed cfc-mtDNA in serum samples of AF patients may originate from tachyarrhythmia in atrial cardiomyocytes.

**Figure 7:**
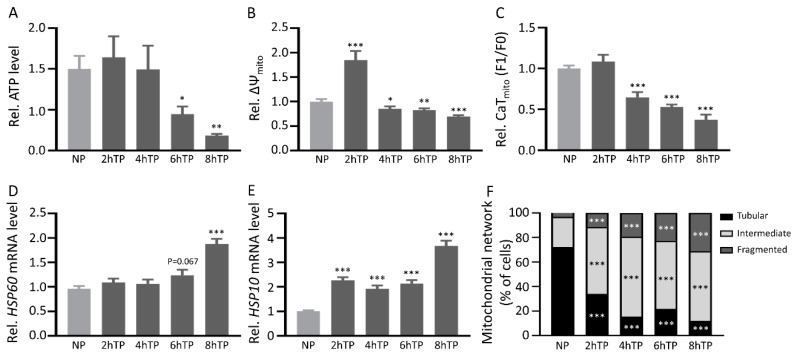
Tachypaced HL-1 cardiomyocytes show mitochondrial damage/dysfunction and stress. Quantified data of (**A**) ATP levels, (**B**) mitochondrial membrane potential, (**C**) mitochondrial calcium transient (CaT_mito_) amplitudes and mitochondrial stress markers (**D**) *HSP60* and (**E**) *HSP10* during NP (normal pacing) or TP (tachypacing) of HL-1 atrial cardiomyocytes. (**F**) Transition of the mitochondrial network from tubular to fragmented from NP to TP. * *p* < 0.05, ** *p* < 0.01, *** *p* < 0.001 vs. NP.

As we previously observed mitochondrial dysfunction to underlie AF in experimental and clinical AF [21], we sought to determine whether mitochondrial damage in the cardiomyocytes is associated with the release of cfc-mtDNA in the medium of tachypaced HL-1 atrial cardiomyocytes. To this end, we evaluated markers of mitochondrial damage/dysfunction (ATP level, mitochondrial membrane potential, amplitude mitochondrial calcium transients, and mitochondrial morphology) and mitochondrial stress (HSP60 and HSP10 mRNA levels) in HL-1 atrial cardiomyocytes, tachypaced for 2, 4, 6, or 8 h (Figure 7). In line with the gradual increase of cfc-mtDNA in the medium, a significant and gradual increase in mitochondrial damage/dysfunction and stress was observed in tachypaced HL-1 atrial cardiomyocytes. These results suggest that the release of cfc-mtDNA is associated with mitochondrial damage, dysfunction, and stress.

## 4. Discussion

This study describes the association of cell-free circulating mitochondrial DNA levels in serum with AF stage and AF recurrence after PVI. Our results indicate that the cfc-mtDNA levels are increased in the early stage of AF (PAF) and decreased in the end-stage of AF (longstanding-persistent AF) in the total study population compared to controls. For the ECV/PVI subgroup of AF patients, this pattern of increase in cfc-mtDNA levels in PAF and decrease in LS-PeAF remained, while no differences in cfc-mtDNA levels were present for AF patients in the cardiac surgery subgroup. Moreover, we demonstrate that there is a difference in cfc-mtDNA levels between males and females and that males, but not females, show increased cfc-mtDNA levels in PAF. Furthermore, we found that cfc-mtDNA levels were associated with recurrence of AF after treatment, especially in PAF in the PVI subgroup. Finally, we showed that the mitochondrial stress-related chaperone HSP60 is not a blood-based marker for AF. However, cfc-mtDNA may be a potential marker, as it is most likely released from cardiomyocytes in the blood upon stress and the circulating levels are increased in PAF. Taken together, our findings suggest that cfc-mtDNA levels may represent a possible interesting serum marker to stage AF and identify patients with PAF, especially male PAF patients who are at risk for AF recurrence after PVI treatment. Future research is warranted to identify the specific applicability of cfc-mtDNA as a diagnostic serum biomarker for AF.

### 4.1. Current Biomarkers in AF

Currently, there is a great need to early detect AF stage and recurrence after treatment. Studies searching for AF serum biomarkers are not new, as several biomarkers, including troponin, brain natriuretic peptide, creatinine, C-reactive protein, and fibroblast growth factor 23, have been associated with AF pathology, progression, and treatment effects [2,9,10,11]. Nevertheless, the use of biomarkers has not been integrated into clinical management of AF due to lack of AF specificity. In most studies, common cardiovascular biomarkers are screened [9]. A disadvantage of this approach is that the targeted biomarkers are not necessarily specific to AF or related to AF pathogenic pathways and therefore may explain the lack of suitability as biomarker candidates for AF staging. Therefore, we sought to target a key modulator of AF pathogenesis. As we showed recently that mitochondrial dysfunction is a key modulator underlying AF in the experimental and clinical AF setting [21], we evaluated cfc-mtDNA as a potential blood-based marker in AF.

### 4.2. Clinical Implications of cfc-mtDNA in AF

In our study, we found in serum samples of patients of the ECV/PVI subgroup that cfc-mtDNA was increased in PAF patients. Moreover, specifically in PAF patients, increased cfc-mtDNA levels were associated with AF recurrence after treatment. Interestingly, cfc-mtDNA is not associated with AF stage in patients in the cardiac surgery subgroup, which may imply that the pathomechanisms and burden of AF in patients treated for AF or for an underlying heart disease may be different. We also evaluated sex differences in our study population and found that the stage of AF can be predicted especially in male compared to female patients. Although the incidence of AF is higher in males, female patients are more symptomatic, have higher heart rates during AF episodes, have a higher chance of recurrence after treatment, and have a higher risk of stroke and mortality than male patients [37,38]. It has been speculated that the fluctuations in female hormones may be (partly) responsible for the difference between male and female patients [38,39]. However, as the women included in this study are in the menopause, other, yet undetermined, factors play a role in the differences in the cfc-mtDNA levels between male and female AF patients. These sex differences imply that cfc-mtDNA may only be an applicable marker for male AF patients, that are treated for AF. Further research in larger patient populations is necessary in order to elucidate the differences between cfc-mtDNA levels in male and female AF patients and their predictive value in AF onset. Nevertheless, the findings in the current study show that pathomechanism-driven biomarker analysis may be advantageous to select novel biomarkers for AF.

### 4.3. The Origin of cfc-mtDNA in Serum

Although it is not exactly known where cfc-mtDNA in the serum originates from, it is possibly released into the circulation during cellular injury, such as apoptosis, necrosis, or trauma [40,41]. As AF is associated with mitochondrial dysfunction and damage [21], this process of mitochondrial stress may result in the release of mitochondrial DNA into the circulation. In addition, (mildly) damaged mitochondria may be incorporated in exosomes, which translocate to the extracellular environment. Here, the mtDNA is released and end up in the circulation, forming cfc-mtDNA [42,43]. The presence of mitochondrial DNA in the media of rapid electrical-stimulated cardiomyocytes confirmed that mitochondrial DNA can be released out of the cardiomyocytes into the circulation. Mitochondrial DNA in the circulation was found to act as a damage-associated pattern (DAMP) [17], due to the bacterial origin of mitochondria and, therefore, may cause an inflammatory response [41]. As inflammation is associated with AF-related pathology [18,20], we may speculate that this is (partly) the cause of the cfc-mtDNA-related DAMPs. Moreover, studies have shown that accumulation of mtDNA damage is associated with additional mitochondrial dysfunction and cardiac pathology [44]. This all implicates that mitochondrial DNA plays an important role in the pathology of AF, which warrants further investigation into the mtDNA-related disease mechanisms. 

### 4.4. Cfc-mtDNA and Remodeling in AF

AF itself may drive the release of mitochondrial DNA into the circulation. As PAF is an early disease stage of AF, one may speculate that episodes of AF in these patients initially induce ATP production in the mitochondria to sustain energy levels during increased electrical activation rates. Indeed, in rapid electrically stimulated cardiomyocytes, early induction of ATP levels was observed [21]. However, as AF leads to mitochondrial dysfunction [21]; this may result in the release of mitochondrial DNA into the circulation. This is in line with findings from an in vivo goat model of AF. Here, goats showed a gradual shift in mitochondrial shape (deformation of mitochondria) until AF became persistent, which coincided with maladaptive and persistent structural damage in mitochondria and atrial cardiomyocytes [45]. Mitochondrial damage may result in exhaustion of mitochondrial function and decreased cfc-mtDNA levels, as observed in PeAF and LS-PeAF patients. As such, mitochondrial damage may represent an early step in response to tachycardia, as the cfc-mtDNA levels were significantly increased in PAF and not PeAF. A comparable pattern, increase in PAF and exhaustion in PeAF and LS-PeAF, has been observed for HSP expression levels in clinical AF [46]. This implicates that mitochondrial DNA release may be a harbinger of the detrimental atrial remodeling seen in AF.

### 4.5. Limitations

Although this study indicates a role for the cfc-mtDNA levels in AF staging and prediction of AF recurrence after treatment, it contains various limitations. One limitation comprises the control patients. These patients have no history of AF and no structural heart disease. However, they do have premature ventricular contractions, Wolff–Parkinson–White syndrome, or are referred for Ajmaline testing. The partial overlap in cfc-mtDNA levels between the control and AF patients may be attributed to the clinical nature of the control patients. Nevertheless, there are significant differences between these two groups, suggesting that cfc-mtDNA is released in a higher amount in the circulation in AF patients than in control patients.

Another limitation is the AF patient population. Unfortunately, the number of patients included in the longstanding-persistent AF group is small, due to limited scheduled cardiac surgeries for this group. Moreover, the number of female patients is lower compared to male patients in all study groups, this is partly due to less willingness of females to participate in the study compared to males. In addition, the classification of AF patients into the different AF stages is difficult, as it is mainly based on electrocardiogram (ECG) measurements, which is inaccurate for AF staging. Although the changes in cfc-mtDNA levels were not outspokenly large between the different ECG-based stages of AF compared to control patients, we nevertheless revealed that cfc-mtDNA levels are significantly increased in PAF and predict recurrence after PVI treatment, especially in PAF patients. Since a recurrence after PVI may be due to an incomplete lesion, and therefore be independent of an underlying substrate (mitochondrial damage), part of the AF recurrence in the PAF population may be false positives. However, the same pattern of increased cfc-mtDNA levels in AF recurrence in PAF patients after ECV was observed, suggesting that the substrate and probably not an incomplete lesion is the cause of AF recurrence after treatment.

## 5. Conclusions

Our study describes for the first time that the level of cfc-mtDNA in serum is associated with AF stage, especially paroxysmal AF. Moreover, the level of cfc-mtDNA is associated with recurrence of AF in patients with paroxysmal AF undergoing AF treatment. In addition, increased levels of cfc-mtDNA in the medium of in vitro electrical stimulated HL-1 cardiomyocytes are associated with enhanced mitochondrial damage and stress in these cardiomyocytes. As clinical AF is associated with mitochondrial damage and stress [21], mtDNA may be released from atrial cardiomyocytes into the circulation due to AF. Future research is warranted to determine the applicability of cfc-mtDNA as a biomarker for AF.

## Figures and Tables

**Table 1:** Primer pairs used for quantitative PCR.

Target Gene	Forward Primer	Reverse Primer
Mouse COX1	GCCCCAGATATAGCATTCCC	GTTCATCCTGTTCCTGCTCC
Mouse ND1	AAACTATGTTCTCCGCCCCAA	TGGAGTCAGTGCATTTTGGC
Mouse 18S rRNA	TAGAGGGACAAGTGGCGTTC	CGCTGAGCCAGTCAGTGT
Human COX3	ATGACCCACCAATCACATGC	ATCACATGGCTAGGCCGGAG
Human ND1	ATACCCATGGCCAACCTCCT	GGGCCTTTGCGTAGTTGTAT
Human ND2 [26]	TAAAACTAGGAATAGCCCCC	TTGAGTAGTAGGAATGCGGT
Human ND3 [26]	CACAACTCAACGGCTACATA	TTGTAGTCACTCATAGGCCA
Human ND4L [26]	AGCATTTACCATCTCACTTCT	GCATTGGAGTAGGTTTAGGTT
Human ND4 [26]	TCTTCTTCGAAACCACACTT	AAGTACTATTGACCCAGCGA
Human ND5 [27]	ACATCTGTACCCACGCCTTC	CAGGGAGGTAGCGATCAGAG
Human ND6 [28]	GTAGGATTGGTGCTGTGG	GGATCCTCCCGAATCAAC
Human ATP8 [29]	CTAAAAATATTAAACACAAACTACCACCTACCTC	GTTCATTTTGGTTCTCAGGGTTTGTTATAA
Human CYB [27]	ACATCGGCATTATCCTCCTG	GTGTGAGGGTGGGACTGTCT
Human 18S rRNA	AGAAACGGCTACCACATCCA	CCCTCCAATGGATCCTCGTT

**Table 2:** Demographic and clinical characteristics of control patients (C), sinus rhythm patients (SR), and atrial fibrillation (AF) patients.

	C	SR	PAF	PeAF	LS-PeAF
N	84	59	100	116	20
Sex					
Male (N, %)	44 (52.4)	47 (79.7) **	73 (73.0) *	87 (75.0) **	16 (80.0) *
Female (N, %)	40 (47.6)	12 (20.3) **	27 (27.0) *	29 (25.0) **	4 (20.0) *
Age (mean ± SD)	50 ± 16	69 ± 11 ***	65 ± 11 ***	65 ± 11 ***	71 ± 9 ***
Underlying heart disease, test (N, %)					
WPW	9 (10.7)	0 (0.0)	0 (0.0)	0 (0.0)	0 (0.0)
PVC	32 (38.1)	0 (0.0)	0 (0.0)	0 (0.0)	0 (0.0)
Ajmaline	43 (51.2)	0 (0.0)	0 (0.0)	0 (0.0)	0 (0.0)
CAD	0 (0.0)	50 (84.7)	7 (7.0)	7 (6.0)	5 (25.0)
AVD	0 (0.0)	14 (23.7)	7 (7.0)	8 (6.9)	6 (30.0)
MVD	0 (0.0)	8 (13.6)	4 (4.0)	12 (10.3)	4 (20.0)
Type procedure					
Cardiac surgery	0 (0.0)	59 (100.0)	15 (15.0)	27 (23.3)	15 (75.0)
PVI	0 (0.0)	0 (0.0)	73 (73.0)	21 (18.1)	2 (10.0)
ECV	0 (0.0)	0 (0.0)	12 (12.0)	68 (58.6)	3 (15.0)
Duration of AF	-	-	97 ± 67	86 ± 62	159 ± 96
(mean±SD (months))					
LA dilatation (> 45 mm, %)	2 (2.4)	13 (22.0) ***	32 (32.0) ***	52 (44.8) ***	13 (65.0) ***
LVF (N, %)					
Normal	68 (81.0)	44 (74.6)	66 (66.0)	70 (60.3) **	12 (60.0)
Mild impairment	9 (10.7)	14 (23.7)	11 (11.0)	29 (25.0) *	7 (35.0) *
Moderate impairment	2 (2.4)	1 (1.7)	4 (4.0)	14 (12.1)	1 (5.0)
Severe impairment	3 (3.6)	0 (0.0)	1 (1.0)	3 (2.6)	0 (0.0)
Medication (N, %)					
ACE inhibitor	23 (27.4)	40 (67.8)	47 (47.0)	57 (49.1)	16 (80.0)
Statin	14 (16.7)	44 (74.6)	40 (40.0)	40 (34.5)	15 (75.0)
Type I AAD	6 (7.1)	1 (1.7)	33 (33.0)	13 (11.2)	1 (5.0)
Type II AAD	26 (31.0)	40 (67.8)	45 (45.0)	63 (54.3)	14 (70.0)
Type III AAD	6 (7.1)	0 (0.0)	45 (45.0)	49 (42.2)	3 (15.0)
Type IV AAD	4 (4.8)	3 (5.1)	4 (4.0)	8 (6.9)	1 (5.0)
Digoxin	1 (1.2)	0 (0.0)	7 (7.0)	21 (18.1)	5 (25.0)
Hypertension (N, %)	21 (25.0)	38 (64.4) ***	52 (52.0) ***	59 (50.9) ***	11 (55.0) *
Diabetes Mellitus (N, %)	5 (6.0)	17 (28.8) ***	11 (11.0)	15 (12.9)	6 (30.0) **
BMI (N, %)					
Underweight (< 18.50)	1 (1.2)	0 (0.0)	0 (0.0)	0 (0.0)	0 (0.0)
Normal (18.5–25)	45 (53.6)	13 (22.0) ***	34 (34.0) *	30 (25.9) ***	3 (15.0) **
Overweight (25–30)	29 (34.5)	26 (44.1)	46 (46.0)	52 (44.8)	10 (50.0)
Obese class I (30–35)	7 (8.3)	17 (28.8) **	17 (17.0)	23 (19.8)	6 (30.0) *
Obese class II (35–40)	2 (2.4)	3 (5.1)	3 (3.0)	10 (8.6)	0 (0.0)
Obese class III (> 40)	0 (0.0)	0 (0.0)	0 (0.0)	1 (0.9)	1 (5.0)

AAD: anti-arrhythmic drug, ACE: angiotensin-converting enzyme, Ajmaline: persons with a family history of Brugada syndrome are tested for this cardiac disease, AVD: aortic valve disease, BMI: body mass index, C: control, CAD: coronary artery disease, ECV: electrical cardioversion, LA: left atrium, LS-PeAF: longstanding persistent AF, LVF: left ventricular function, MVD: mitral valve disease, PAF: paroxysmal AF, PeAF: persistent AF, PVC: premature ventricular contraction, PVI: pulmonary vein isolation, SR: sinus rhythm, WPW: Wolff–Parkinson–White. * *p* < 0.05, ** *p* < 0.01, *** *p* < 0.001 vs. C.

## Supplementary Materials

The following are available online at https://www.mdpi.com/2073-4409/9/5/1159/s1, Figure S1: Scheme about the study population and description of different classifications, Figure S2: No major differences in cell-free circulating mtDNA levels between control and sinus rhythm groups, Figure S3: Additional mtDNA genes show the same trend as COX3 and ND1, Figure S4: Cfc-mtDNA levels in ECV and PVI groups separately, Table S1: Demographic and clinical characteristics of AF patients undergoing open-heart surgery, Table S2: Demographic and clinical characteristics of AF patients treated for AF by electrical cardioversion (ECV) or pulmonary vein isolation (PVI), Table S3: Demographic and clinical characteristics of AF patients treated for AF by electrical cardioversion (ECV), Table S4: Demographic and clinical characteristics of AF patients treated for AF by pulmonary vein isolation (PVI), Table S5: Mean mtDNA levels of COX3 and ND1 for each group.
