# Supplementary material for: Cell-Free Circulating Mitochondrial DNA: A Potential Blood-Based Marker for Atrial Fibrillation
Source: Cells. 2020 May 8;9(5):1159. doi: 10.3390/cells9051159 (PMC7290331; doi:10.3390/cells9051159)
Supplement: Supplementary file 1 [file cells-09-01159-s001.pdf]

SUPPLEMENTAL MATERIAL

Supplemental figures

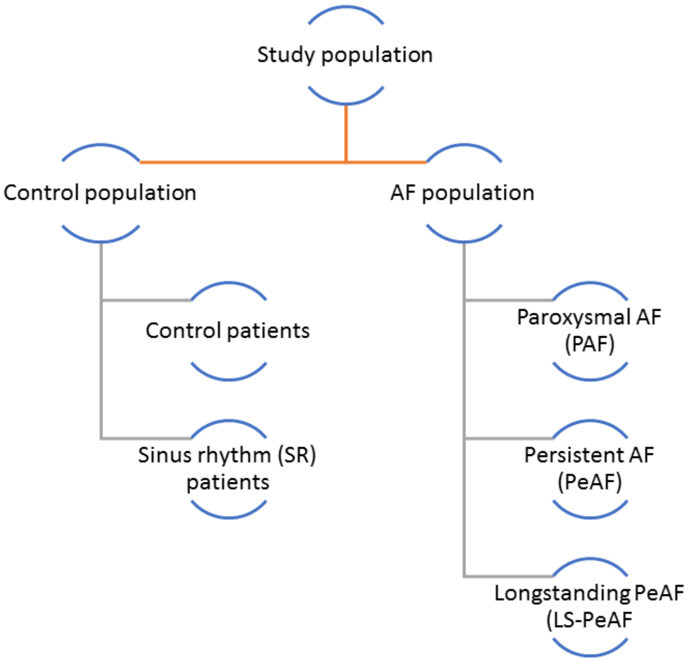

Control patients	• No history of AF<br>• No structural heart disease<br>• Either:<br>• Premature ventricular contraction<br>• Wolff-Parkinson-White syndrome<br>• Referred for Ajmaline testing
SR patients	• No history of AF<br>• Underlying cardiovascular disease
PAF	AF episodes last from minutes to less than a week
PeAF	AF episodes last longer than a week
LS-PeAF	AF last more than one year

Figure S1 Scheme about the study population and description of different classifications.

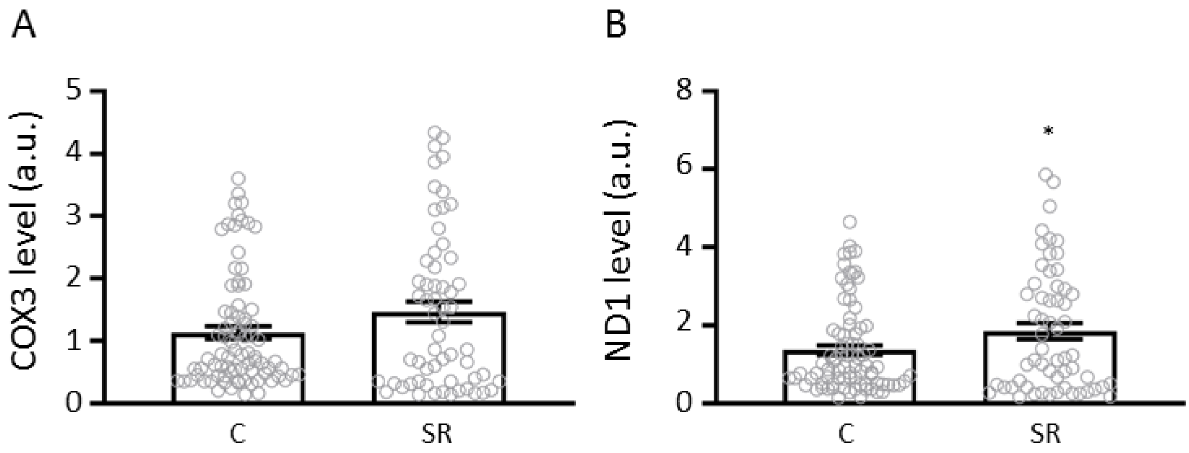

Figure S2 No major differences in cell-free circulating mtDNA levels between control and sinus rhythm groups. Cfc-mtDNA levels for **A)** COX3 and **B)** ND1 in control (C) patients and in sinus rhythm (SR) patients. Only ND1 shows a minor increase in SR. **P*<0.05 vs C.

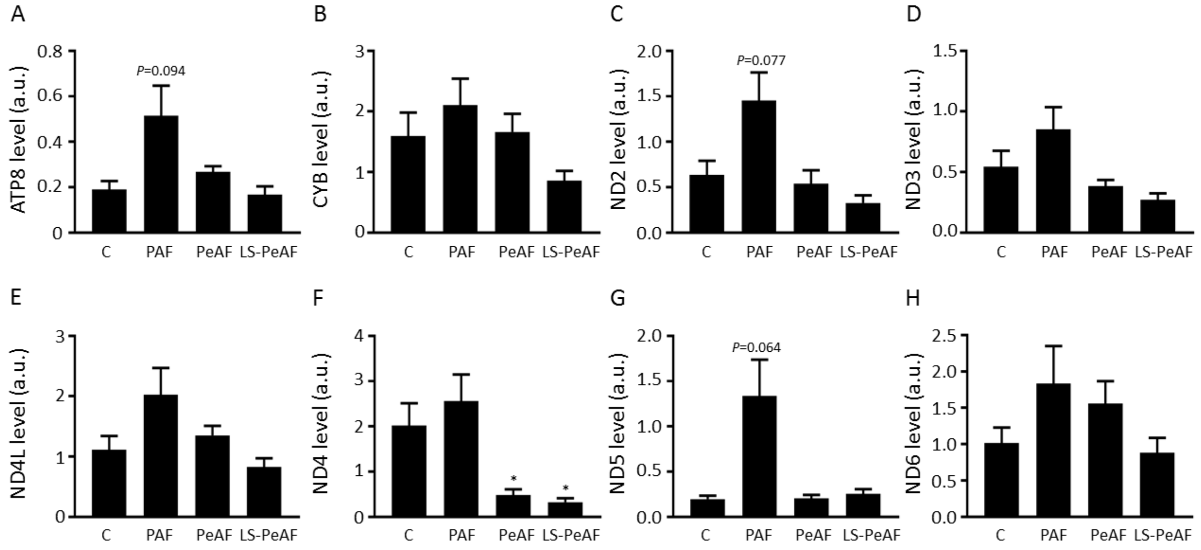

Figure S3 Additional mtDNA genes show the same trend as COX3 and ND1. Cfc-mtDNA levels of 8 additional mtDNA genes were determined in a small study of 10 patients per group. C: control patients, PAF: paroxysmal AF, PeAF: persistent AF, LS-PeAF: longstanding-persistent AF, ATP8: ATP synthase, subunit 8, CYB: cytochrome B, ND2: NADH dehydrogenase, subunit 2, ND3: NADH dehydrogenase, subunit 3, ND4L: NADH dehydrogenase, subunit 4L, ND4: NADH dehydrogenase, subunit 4, ND5: NADH dehydrogenase, subunit 5, ND6: NADH dehydrogenase, subunit 6. *P<0.05 vs C.

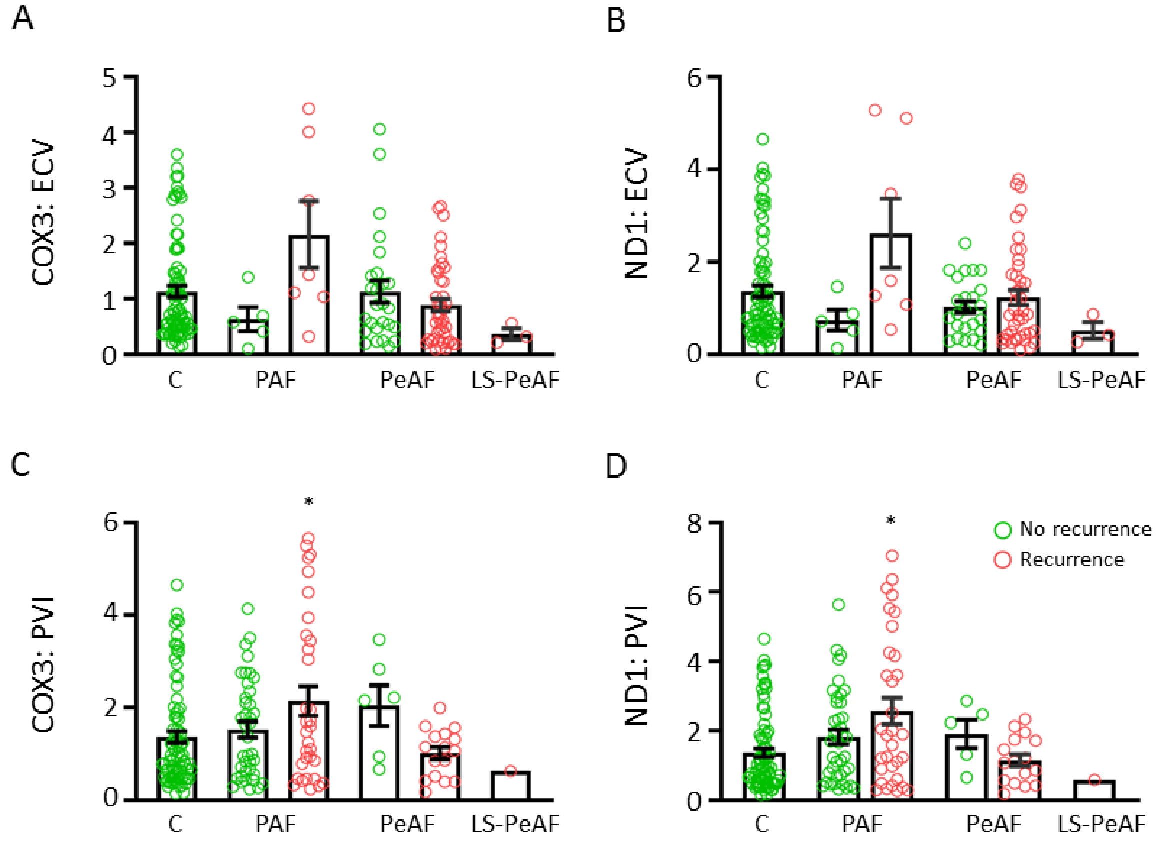

Figure S4 Cfc-mtDNA levels in ECV and PVI groups separately. Cfc-mtDNA levels for the ECV group for **A)** COX3 and **B)** ND1 and for the PVI group for **C)** COX3 and **D)** ND1 in control patients and in patients with different AF stages that are treated for AF. C: control, PAF: paroxysmal AF, PeAF: persistent AF, LS-PeAF: longstanding-persistent AF. *P<0.05

Supplemental tables

Table S1 Demographic and clinical characteristics of AF patients undergoing open-heart surgery

	PAF	PeAF	LS-PeAF
N	15	27	15
Sex			
Male (N,%)	10 (66.7)	19 (70.4)	12 (80.0)
Female (N,%)	5 (33.3)	8 (29.6)	3 (20.0)
Age (mean±SD)	71±15	70±9	75±6
Underlying heart disease (N,%)			
CAD	7 (46.7)	7 (25.9)	5 (33.3)
AVD	7 (46.7)	8 (29.6)	5 (33.3)
MVD	4 (26.7)	12 (44.4)	4 (26.7)
Duration of AF (mean±SD (months))	80±92	66±51	163±104
LA dilatation (>45mm,%)	5 (33.3)	16 (59.3)	12 (80.0)
LVF (N,%)			
Normal	12 (80.0)	16 (59.3)	9 (60.0)
Mild impairment	2 (13.3)	5 (18.5)	5 (33.3)
Moderate impairment	1 (6.7)	5 (18.5)	1 (6.7)
Severe impairment	0 (0.0)	1 (3.7)	0 (0.0)
Medication (N,%)			
ACE inhibitor	8 (53.3)	17 (63.0)	13 (86.7)
Statin	9 (60.0)	9 (33.3)	11 (73.3)
Type I AAD	2 (13.3)	1 (3.7)	0 (0.0)
Type II AAD	9 (60.0)	19 (70.4)	13 (86.7)
Type III AAD	4 (26.7)	4 (14.8)	1 (6.7)
Type IV AAD	0 (0.0)	1 (3.7)	1 (6.7)
Digoxin	1 (6.7)	7 (25.9)	4 (26.7)
Hypertension (N,%)	10 (66.7)	16 (59.3)	8 (53.3)
Diabetes Mellitus (N,%)	2 (13.3)	4 (14.8)	5 (33.3)
BMI (N,%)			
Underweight (<18.50)	0 (0.0)	0 (0.0)	0 (0.0)
Normal (18.5-25)	7 (46.7)	8 (29.6)	2 (13.4)
Overweight (25-30)	6 (40.0)	11 (40.8)	8 (53.3)
Obese class I (30-35)	2 (13.3)	4 (14.8)	5 (33.3)
Obese class II (35-40)	0 (3.0)	4 (14.8)	0 (0.0)
Obese class III (>40)	0 (0.0)	0 (0.0)	0 (0.0)

Table S2 Demographic and clinical characteristics of AF patients treated for AF by electrical cardioversion (ECV) or pulmonary vein isolation (PVI)

	PAF	PeAF	LS-PeAF
N	85	89	5
Sex			
Male (N,%)	63 (74.1)	68 (76.4)	4 (80.0)
Female (N,%)	22 (25.9)	21 (23.6)	1 (20.0)
Age (mean±SD)	64±10	63±11	60±9
Duration of AF (mean±SD (months))	100±62	91±64	149±78
LA dilatation (>45mm,%)	27 (31.8)	36 (40.4)	1 (20.0)
LVF (N,%)			
Normal	63 (74.1)	54 (60.7)	3 (60.0)
Mild impairment	9 (10.9)	24 (27.0)	2 (20.0)
Moderate impairment	3 (3.5)	9 (10.1)	0 (0.0)
Severe impairment	1 (1.2)	2 (2.2)	0 (0.0)
Medication (N,%)			
ACE inhibitor	39 (45.9)	40 (44.9)	3 (60.0)
Statin	31 (36.5)	31 (34.8)	4 (80.0)
Type I AAD	31 (36.5)	12 (13.5)	1 (20.0)
Type II AAD	36 (42.4)	44 (49.4)	1 (20.0)
Type III AAD	41 (48.2)	45 (50.6)	2 (20.0)
Type IV AAD	4 (4.7)	7 (7.9)	0 (0.0)
Digoxin	6 (7.1)	14 (15.7)	1 (20.0)
Hypertension (N,%)	42 (49.4)	43 (48.3)	3 (60.0)
Diabetes Mellitus (N,%)	9 (10.9)	11 (12.4)	1 (20.0)
BMI (N,%)			
Underweight (<18.50)	0 (0.0)	0 (0.0)	0 (0.0)
Normal (18.5-25)	27 (31.8)	22 (24.7)	1 (20.0)
Overweight (25-30)	40 (47.1)	41 (46.1)	2 (40.0)
Obese class I (30-35)	15 (17.6)	19 (21.3)	1 (20.0)
Obese class II (35-40)	3 (3.5)	6 (6.7)	0 (0.0)
Obese class III (>40)	0 (0.0)	1 (1.2)	1 (20.0)

Tabel S3 Demographic and clinical characteristics of AF patients treated for AF by electrical cardioversion (ECV)

	PAF	PeAF	LS-PeAF
N	12	68	3
Sex			
Male (N,%)	10 (83.3)	54 (79.4)	3 (100.0)
Female (N,%)	2 (16.7)	14 (20.6)	0 (0.0)
Age (mean±SD)	65±11	63±12	62±7
Duration of AF (mean±SD (months))	118±69	89±69	154±110
LA dilatation (>45mm,%)	6 (50.0)	27 (39.7)	0 (0.0)
LVF (N,%)			
Normal	10 (83.3)	38 (55.9)	2 (66.7)
Mild impairment	0 (0.0)	20 (29.4)	1 (33.3)
Moderate impairment	1 (8.3)	8 (11.8)	0 (0.0)
Severe impairment	1 (8.3)	2 (2.9)	0 (0.0)
Medication (N,%)			
ACE inhibitor	9 (75.0)	32 (47.1)	1 (33.3)
Statin	5 (41.7)	26 (38.2)	2 (66.7)
Type I AAD	2 (16.7)	6 (8.8)	1 (33.3)
Type II AAD	7 (58.3)	35 (51.5)	0 (0.0)
Type III AAD	3 (25.0)	32 (47.1)	1 (33.3)
Type IV AAD	3 (25.0)	2 (2.9)	0 (0.0)
Digoxin	1 (8.3)	10 (14.7)	1 (33.3)
Hypertension (N,%)	9 (75.0)	31 (45.6)	1 (33.3)
Diabetes Mellitus (N,%)	1 (8.3)	10 (14.7)	0 (0.0)
BMI (N,%)			
Underweight (<18.50)	0 (0.0)	0 (0.0)	0 (0.0)
Normal (18.5-25)	2 (16.7)	18 (26.5)	1 (33.3)
Overweight (25-30)	8 (66.7)	30 (44.1)	2 (66.7)
Obese class I (30-35)	1 (8.3)	15 (22.1)	0 (0.0)
Obese class II (35-40)	1 (8.3)	4 (5.9)	0 (0.0)
Obese class III (>40)	0 (0.0)	1 (1.5)	0 (0.0)

Table S4 Demographic and clinical characteristics of AF patients treated for AF by pulmonary vein isolation (PVI)

	PAF	PeAF	LS-PeAF
N	73	21	2
Sex			
Male (N,%)	53 (72.6)	14 (66.7)	1 (50.0)
Female (N,%)	20 (27.4)	7 (33.3)	1 (50.0)
Age (mean±SD)	64±10	64±9	56±12
Duration of AF (mean±SD (months))	97±61	99±46	141±17
LA dilatation (>45mm,%)	21 (28.8)	9 (42.9)	1 (50.0)
LVF (N,%)			
Normal	62 (84.9)	16 (76.2)	1 (50.0)
Mild impairment	9 (12.3)	4 (19.0)	1 (50.0)
Moderate impairment	2 (2.8)	1 (4.8)	0 (0.0)
Severe impairment	0 (0.0)	0 (0.0)	0 (0.0)
Medication (N,%)			
ACE inhibitor	30 (41.1)	8 (38.1)	2 (100.0)
Statin	26 (35.6)	5 (23.8)	2 (100.0)
Type I AAD	29 (39.7)	6 (28.6)	0 (0.0)
Type II AAD	29 (39.7)	9 (42.9)	1 (50.0)
Type III AAD	38 (52.1)	13 (61.9)	1 (50.0)
Type IV AAD	1 (1.4)	5 (23.8)	0 (0.0)
Digoxin	5 (6.8)	4 (19.0)	0 (0.0)
Hypertension (N,%)	33 (45.2)	12 (57.1)	2 (100.0)
Diabetes Mellitus (N,%)	8 (11.0)	1 (4.8)	1 (50.0)
BMI (N,%)			
Underweight (<18.50)	0 (0.0)	0 (0.0)	0 (0.0)
Normal (18.5-25)	25 (34.2)	4 (19.0)	0 (0.0)
Overweight (25-30)	32 (43.8)	11 (52.5)	0 (0.0)
Obese class I (30-35)	14 (19.2)	4 (19.0)	1 (50.0)
Obese class II (35-40)	2 (2.8)	2 (9.5)	0 (0.0)
Obese class III (>40)	0 (0.0)	0 (0.0)	1 (50.0)

Table S5 Mean mtDNA levels of COX3 and ND1 for each group

	COX3				ND1			
	C+SR	PAF	PeAF	LS-PeAF	C+SR	PAF	PeAF	LS-PeAF
Cardiac surgery +ECV/PVI	1.27	1.75	0.99	0.76	1.56	1.97	1.26	1.01
	C	PAF	PeAF	LS-PeAF	C	PAF	PeAF	LS-PeAF
ECV/PVI	1.13	1.85	0.96	0.43	1.36	2.05	1.20	0.53
ECV	1.13	1.52	0.89	0.36	1.36	1.83	1.16	0.52
PVI	1.13	1.91	1.19	0.62	1.36	2.08	1.33	0.58
	SR	PAF	PeAF	LS-PeAF	SR	PAF	PeAF	LS-PeAF
Cardiac surgery	1.46	0.98	1.08	0.82	1.85	1.26	1.45	1.09
